# Anterior gradient 2 increases long-chain fatty acid uptake via stabilizing FABP1 and facilitates lipid accumulation

**DOI:** 10.7150/ijbs.57099

**Published:** 2021-02-08

**Authors:** Yunqiu Wang, Mengqi Jia, Chuanjie Liang, Nan Sheng, Xiaodan Wang, Fang Wang, Yanhai Luo, Jin Jiang, Liangyu Cai, Huanmin Niu, Deyu Zhu, Effat Un Nesa, Charles YF Young, Huiqing Yuan

**Affiliations:** 1 Key Laboratory of Experimental Teratology of Ministry of Education, Institute of Medical Sciences, the Second Hospital, Cheeloo College of Medicine, Shandong University, Jinan 250031, China.; 2 Department of Biochemistry and Molecular Biology, School of Basic Medical Sciences, Cheeloo College of Medicine, Shandong University, Jinan 250014, China.; 3 Department of Urology, Mayo Clinic College of Medicine, Mayo Clinic, Rochester 55905, MN, USA.

**Keywords:** AGR2, lipid metabolism, chaperone, FABP1, liver, intestine

## Abstract

Anterior gradient 2 (AGR2), a protein disulfide isomerase (PDI), is a well-established oncogene. Here, we found that *Agr2^-/-^* mice had a decreased fat mass and hepatic and serum lipid levels compared with their wild-type littermates after fasting, and exhibited reduced high-fat diet (HFD)-induced fat accumulation. Transgenic mice overexpressing AGR2 (*Agr2*/Tg) readily gained fat weight on a HFD but not a normal diet. Proteomic analysis of hepatic samples from *Agr*2^-/-^ mice revealed that depletion of AGR2 impaired long-chain fatty acid uptake and activation but did not affect *de novo* hepatic lipogenesis. Further investigations led to the identification of several effector substrates, particularly fatty acid binding protein-1 (FABP1) as essential for the AGR2-mediated effects. AGR2 was coexpressed with FABP1, and knockdown of AGR2 resulted in a reduction in FABP1 stability. Physical interactions of AGR2 and FABP1 depended on the PDI motif in AGR2 and the formation of a disulfide bond between these two proteins. Overexpression of AGR2 but not a mutant AGR2 protein lacking PDI activity suppressed lipid accumulation in cells lacking FABP1. Moreover, AGR2 deficiency significantly reduced fatty acid absorption in the intestine, which might be resulted from decreased fatty acid transporter CD36 in mice. These findings demonstrated a novel role of AGR2 in fatty-acid uptake and activation in both the liver and intestine, which contributed to the AGR2-mediated lipid accumulation, suggesting that AGR2 is an important regulator of whole-body lipid metabolism and down-regulation of AGR2 may antagonize the development of obesity.

## Introduction

The endoplasmic reticulum (ER) is an organelle important for controlling protein quality, calcium metabolism, lipid synthesis and protein posttranslational modification and trafficking 1-6. ER-resident proteins, such as chaperones, stress response proteins, and calcium-modulating proteins, are critical in maintaining ER homeostasis and cell function 7-9. Protein disulfide isomerases (PDIs) are isomerases and molecular chaperones localized primarily within the ER 10. PDIs possess disulfide interchange and/or oxidoreductase activity via distinct motifs, in which protein disulfide bonds are oxidized, reduced or isomerized 11. As the rate-controlling catalysts, PDIs promote protein folding and the correction of misfolded proteins into their native conformations via their chaperone and/or isomerase activity, functioning as critical factors in the regulation of stability and activity of diverse substrate proteins and maintenance proteostasis 12, 13. Hence, dysfunction of these enzymes has been considered to contribute to the development of diseases, including neurodegeneration, cancer, and cardiovascular diseases 14, 15. However, the role of PDIs in metabolic disorders including dyslipidemia remains largely unknown.

As most lipid metabolic enzymes contain one or more cysteine residues that are essential for maintaining their protein conformation and exerting function, PDIs are emerging as important regulators in lipid metabolism. For example, the PDI family member ERdj5 and PDI/PDIA1 are required for efficient folding of the LDL receptor and apoB100, facilitating very low density lipoprotein (VLDL) assembly 16. ERp44 cooperates with Ero1α to control the quality of adiponectin via thiol-mediated retention 17.

In the present study, we showed a previously unrecognized function of anterior gradient 2 (AGR2), a member of the PDI family which is a well-established oncogenic and prometastatic factor in most adenocarcinomas 18, 19, in lipid metabolism. AGR2 is highly expressed in the liver and intestine but not in adipose tissue, AGR2 null mice (*Agr2*^-/-^) lost fat mass and fat storage, associated with a reduction in blood lipid levels. Further investigations demonstrated the importance of AGR2 in mediating long-chain fatty acid (LCFA) uptake/activation and absorption in the liver and intestine. This effect is ascribed to the AGR2 chaperone activity in maintaining substrate stability.

## Materials and Methods

### Animals

C57BL/6J mice (6-week-old) were obtained from Vital River Laboratory Animal Technology Co. (Beijing, China). *Agr2*^-/-^ mice were obtained from The Jackson Laboratory (USA). TgTn (CAG-*Agr2*-WPRE-pA) overexpression transgenic mice (*Agr2*/Tg) were obtained from Shanghai Model Organisms Center (Shanghai, China) using the promoter CAG. All experiments used male mice of the same age. Mice were caged in an environment with controlled temperature and humidity with free access to water and food under a 12-h light/dark cycle. At the end of the experiments, the mice were euthanized with an overdose of pentobarbital sodium (150 mg/kg, i.v.). All animal experiments were approved by the Ethics Committee of Shandong University School of Medicine (Jinan, China).

### HFD-induced obese mice

Diets were purchased from Trophic Animal Feed High-tech Co. (Nantong, China). After 1 week of diet adaptation, the mice at 8-week-old were fed a HFD (60% fat) for an additional 10 weeks to induce obesity. Mice fed a NCD were used as lean controls.

### Co-immunoprecipitation

Aliquots of proteins (1.2 mg) from cells were precleared with protein A/G Plus-Agarose (Santa Cruz) in the presence of non-specific IgG (Santa Cruz), then incubated with primary antibodies and 20 µl agarose beads with continuous mixing overnight at 4 °C. The beads were washed and heated at 75 °C for 5 min in loading buffer prior to immunoblotting assays.

### Body fat analysis

The body fat mass of mice was analyzed with a LUNAR Prodigy X-Ray Tube Housing Assembly (GE Medical Systems).

### Lipid absorption in mice

Mice were fasted for 4 h and then administered a 100 μL volume of 1.5 mg/kg body weight BODIPY FL-C12 (a FA analog; AAT Bioquest; resuspended in DMSO) by oral gavage. BODIPY is an optimal lipid used for studying mucosal absorption. Additionally, the BODIPY fluorophore itself is intrinsically lipophilic, and probes incorporating this fluorophore undergo native-like transport and metabolism in cells. Serum BODIPY FL-C12 concentrations were measured in blood serially drawn 50, 100 and 150 min after gavage and were quantified by measuring the fluorescence intensity at an excitation wavelength of 488 nm and an emission wavelength of 515 nm 20.

### Histidine (His) pull-down experiments

In His pull-down assays, about 50μg of the appropriate 6×His fusion proteins was mixed with 500μL of the *in vitro* translated products and incubated in binding buffer (20 mM Tris buffer PH 8.0, 150 mM NaCl). The binding reaction was then added to 30 μL of Co-Sepharose beads and mixed at 4 °C for 2 h. The beads were washed three times with washing buffer (20 mM Tris buffer PH 8.0, 150 mM NaCl, 10 mM Imidazole), resuspended in 50 μL of Elution buffer (20 mM Tris-HCl buffer pH 8.0, 150 mM NaCl, 250 mM Imidazole), and resolved on 13.5% gels. Protein bands were detected with Coomassie staining and western blotting.

### Quantitative proteomic analysis by TMT technology

Livers from 8-week-old WT and *Agr2*^-/-^ mice were collected into 2mL tubes, and SDT buffer was added to the sample. The lysate was homogenized in an MP homogenizer (24×2; two times at 6.0 M/S for 60 s each). The homogenate was sonicated and was then boiled for 15 min. After centrifugation at 14000 × g for 40 min, the supernatant was filtered through 0.22 µm filters. The protein content in the filtrate was quantified with a BCA Protein Assay Kit (Bio-Rad, USA). The sample was stored at -80 °C after repeated ultrafiltration (Microcon units, 10 kD). Then, 200 μg of protein per sample was digested with 4 μg trypsin (Promega). The resulting peptides were collected as a filtrate, and the peptide content was estimated by the UV light spectral density at 280 nm. A 100 μg peptide mixture of each sample was labeled using TMT reagent according to the manufacturer's instructions (Thermo Fisher Scientific). LC-MS/MS analysis was performed over a 60 min period on a Q Exactive mass spectrometer (Thermo Scientific) coupled to an Easy nLC system (Proxeon Biosystems, now Thermo Fisher Scientific). MS/MS spectra were searched using the MASCOT engine (Matrix Science, London, UK; version 2.2) embedded into Proteome Discoverer 1.4.

### Bioinformatic analysis

To further explore the impact of differentially expressed proteins on cell physiological processes and discover internal relations between differentially expressed proteins, enrichment analysis was performed. GO enrichment analysis in three ontologies (biological process, molecular function, and cellular component) and KEGG pathway enrichment analysis were applied based on Fisher's exact test, considering all quantified protein annotations as a background dataset. The Benjamini-Hochberg correction for multiple testing was further applied to adjust derived p-values. Only functional categories and pathways with p-values less than the threshold of 0.05 were considered significant. In general, p-values less than the threshold of 0.05 were considered to indicate significant differences. The relative expression data for the studied proteins were used to perform hierarchical clustering analysis. For this purpose, Cluster 3.0 and Java Treeview software were used. The Euclidean distance algorithm for similarity measurement and the average linkage clustering algorithm (clustering is performed using the centroids of the observations) for clustering were selected for hierarchical clustering. Heatmaps are often presented as visual aids in addition to dendrograms. The protein-protein interaction (PPI) information for the studied proteins was retrieved from STRING software. The results were downloaded in XGMML format and imported into Cytoscape 3.7.1 software. Docking analysis was performed by in silico prediction of protein binding using AutoDock Vina.

### Isolation and culture of primary mouse hepatocytes

Mice were anesthetized with chloral hydrate; the livers were perfused with normal saline for 5 min, and *in vivo* digestion was performed with collagenase (900 mg/L) for 5 min. Hepatocytes were separated by centrifugation for 5 min at 300 rpm, washed in PBS and plated on collagen-coated plates. Primary mouse hepatocytes were maintained in low-glucose DMEM (HyClone, Logan, USA) supplemented with 20% fetal bovine serum, 5 mg/L insulin, 5 mg/L transferrin, 20 μg/L dexamethasone and 2 nM glutamine 21.

### Statistical analysis

The data are presented as the mean ± SD and analyzed with GraphPad Prism software (GraphPad, La Jolla, CA, USA). Student's t-test or one-way ANOVA analysis was used for comparison among different groups. All the experiments have been repeated at least three times. Values of P < 0.05 was considered to be statistically significant, indicated as *P < 0.05, **P < 0.01, and ***P < 0.001 in the figures.

Further experimental information is described in the supplementary materials and methods.

## Results

### AGR2 knockout reduces serum lipid levels and fat accumulation

**Figure 1:**
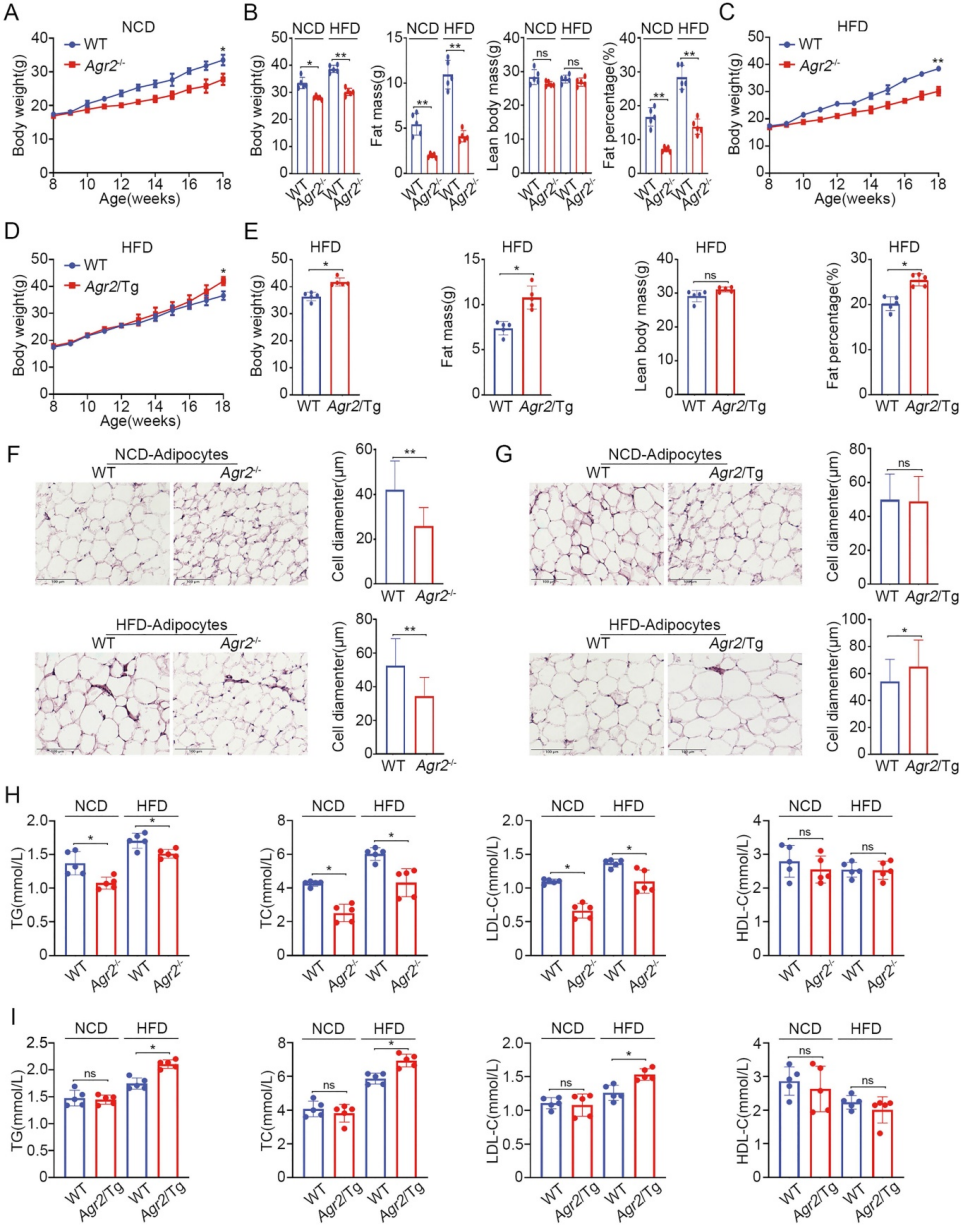
** AGR2 knockout reduces serum lipid levels and fat accumulation.** The 8-week-old mice were fed a NCD or a HFD for an additional 10 weeks. **A.** Body weight of WT and *Agr2*^-/-^ mice during the 10 weeks of NCD feeding (n=5). **B.** Body weight, fat mass, lean body mass and fat percentage of WT and *Agr2*^-/-^ mice fed a NCD or a HFD (n=5). **C.** Body weight of WT and *Agr2*^-/-^ mice during the 10 weeks of HFD feeding (n=5). **D.** Body weight of WT and *Agr2*/Tg mice during the 10 weeks of HFD feeding (n=5). **E.** Body weight, fat mass, lean body mass and fat percentage of WT and *Agr2*/Tg mice fed a HFD (n=5). **F.** Adipose tissue staining and the diameter of adipocytes in WT and *Agr2*^-/-^ mice fed a NCD (top) or a HFD (bottom). **G.** Adipose tissue staining and the diameter of adipocytes in WT and *Agr2*/Tg mice fed NCD (top) or a HFD (bottom).** H.** Circulating lipid profiles of WT and *Agr2*^-/-^ mice fed a NCD or a HFD (n=5). **I.** Circulating lipid profiles of WT and *Agr2*/Tg mice fed a NCD or a HFD (n=5). Representative figures were generated with data from at least three independent experiments. The data are presented as the mean ± SD values. *P < 0.05, **P < 0.01 by Student's t test.

We initially investigated the physiological role of AGR2 using *Agr2*^-/-^ mice 22. It was observed that weight gain was significantly decelerated in *Agr2*^-/-^ mice compared with their wild-type (WT) littermates fed a normal chow diet (NCD) after 10 weeks, and *Agr2*^-/-^ mice weighed 17% less than their WT littermates for 18 weeks (Figure 1A and Figure S1A-C). Whole body composition analysis by dual energy X-ray absorptiometry (DEXA) indicated that *Agr2*^-/-^ mice had a proportionally lower fat mass and that the fat percentage (as a fraction of total body weight) was decreased by 64% at 18-week compared to that of the paired WT mice, but the body weight of* Agr2*^-/-^ and WT mice was comparable when the fat weight was subtracted from the total weight (Figure 1B). AGR2 depletion did not alter food or water intake, eliminating the possibility that the leanness resulted from a lower food intake (Figure S1D, S1E). No significant differences were found in the liver, kidney, heart, spleen, and lung weights or the tibia length between *Agr2*^-/-^ and WT mice fed a NCD. However, the liver and spleen weights were increased in* Agr2*^-/-^ mice fed a HFD, although the weights of the other organs were unchanged (Figure S1F, S1G). Importantly, mice lacking AGR2 showed partial resistance to HFD-induced overweight, and the total fat mass was decreased by 60% in *Agr2*^-/-^ mice compared to the matched WT mice, but no significant change was observed in the fat-free body weight between *Agr2*^-/-^ mice and WT mice (Figure 1B, 1C). Meanwhile, the mass of epididymal white adipose tissue (eWAT) and subcutaneous white adipose tissue (subWAT) was significantly reduced in *Agr2*^-/-^ mice fed either a NCD or a HFD, the ratio of eWAT/whole body weight and subWAT/whole body weight was also markedly decreased in *Agr2*^-/-^ mice after NCD or HFD feeding, the change pattern was similar to observations on whole body fat (Figure S1H, S1I). These findings suggest that AGR2 depletion may reduce fat accumulation. We further assessed phenotypic changes in transgenic mice with AGR2 overexpression (*Agr2*/Tg mice) (Figure S2A-D). Compared to the corresponding WT littermates, *Agr2*/Tg mice exhibited a noticeably increased fat weight gain or percent body fat when fed a HFD but not when fed a NCD (Figure 1D, 1E and Figure S2E-H), demonstrating that AGR2 facilitates fat deposition upon HFD feeding.

We further analyzed the changes in adipocytes (epididymal white adipose tissue) in genetically modified mice. The immunostaining results showed that the adipocytes in *Agr2*^-/-^ mice were much smaller than those in WT mice fed either a NCD or a HFD (Figure 1F). By contrast, the adipocyte diameter was increased in *Agr2*/Tg mice fed a HFD (Figure 1G), supporting the observations that AGR2 exerts a regulatory effect on fat metabolism.

Since AGR2 is not expressed in adipocytes, we next determined whether the AGR2-mediated effect on fat deposits has resulted from alterations in serum lipid levels. The results clearly showed that loss of AGR2 efficiently reduced plasma triglyceride (TG), total cholesterol (TC) and low-density lipoprotein cholesterol (LDL-C) levels (Figure 1H). TG levels decreased by 23% and 12%, TC levels by 42% and 27%, and LDL-C levels by 41% and 22% in response to NCD and HFD feeding, respectively (Figure 1H). However, AGR2 depletion did not affect high-density lipoprotein cholesterol (HDL-C) levels (Figure 1H). By contrast, plasma TG, TC, and LDL-C levels were enhanced but the HDL-C level remained almost unchanged in *Agr2*/Tg mice under HFD feeding conditions (Figure 1I). Notably, plasma lipid levels were not affected in *Agr2*/Tg mice fed a NCD (Figure 1I), implying that a high level of AGR2 was unable to enhance lipid metabolism under NCD feeding conditions. Thus, AGR2 may regulate lipid metabolism, which in turn affects fat storage.

### AGR2 deficiency impairs hepatic lipid synthesis

**Figure 2:**
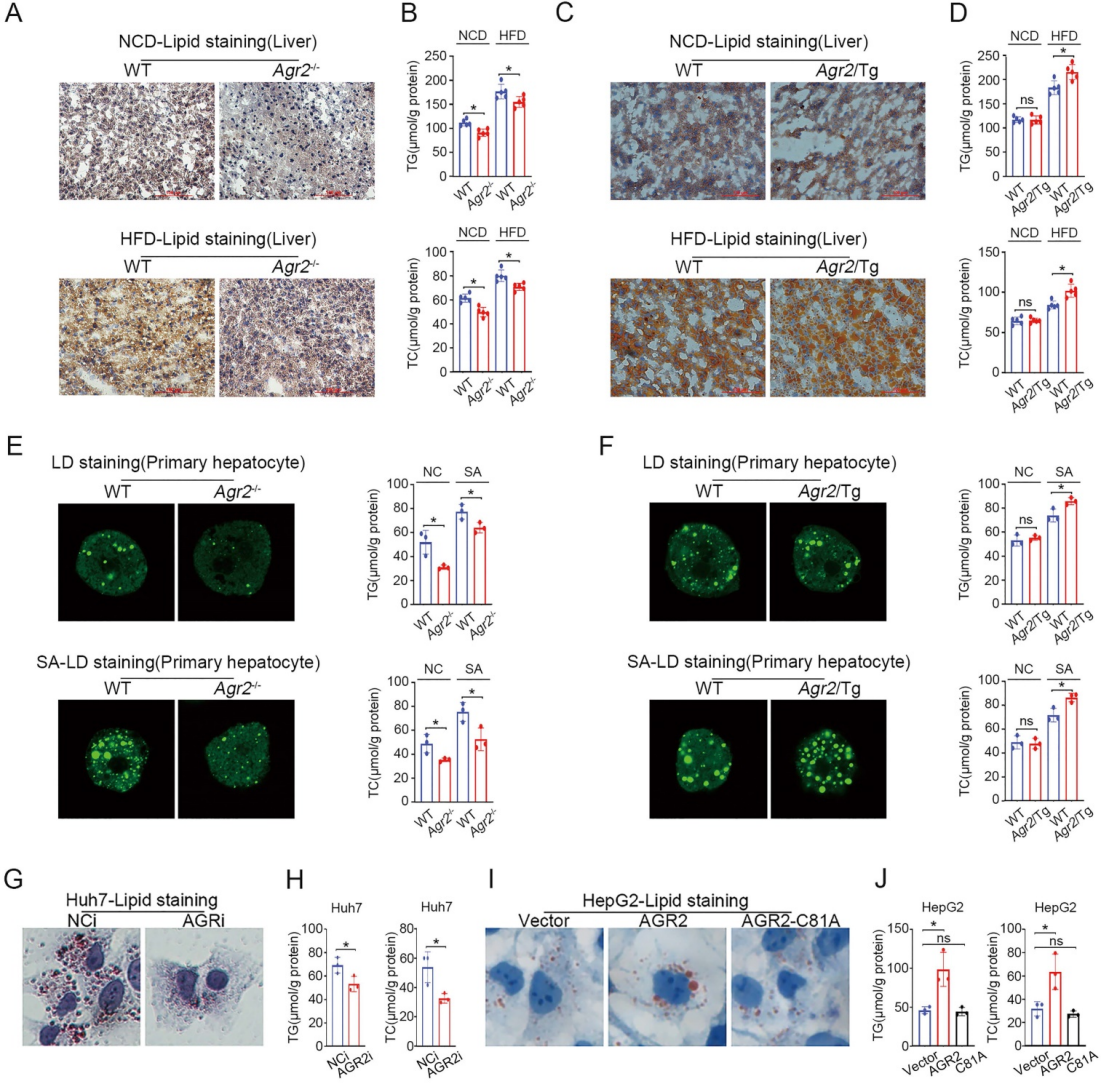
**AGR2 promotes hepatic lipid synthesis.** The 8-week-old mice were fed a NCD or a HFD for an additional 10 weeks. **A.** Lipid staining in liver tissue from WT and *Agr2^-/-^* mice fed a NCD (top) or a HFD (bottom). **B.** TG (top) and TC (bottom) contents in the livers of WT and *Agr2^-/-^* mice fed a NCD or a HFD (n=5). **C.** Lipid staining in liver tissue from WT and *Agr2*/Tg mice fed a NCD (top) or a HFD (bottom). **D.** TG (top) and TC (bottom) contents in the livers of WT and *Agr2*/Tg mice fed a NCD or a HFD (n=5). **E.** Lipid droplet staining (left) and TG and TC contents (right) in primary hepatocytes in WT and *Agr2^-/-^* mice under normal conditions or under exposure to stearic acid. **F.** Lipid droplet staining (left) and TG and TC (right) in primary hepatocytes in WT and *Agr2*/Tg mice under normal conditions or under exposure to stearic acid. **G.** Lipid staining in Huh7 cells treated with siRNA targeting AGR2. **H.** TG and TC contents in Huh7 cells treated with siRNA targeting AGR2. **I.** Lipid staining in HepG2 cells treated with AGR2 and AGR2-C81A expression plasmids.** J.** TG and TC contents in HepG2 cells treated with AGR2 and AGR2-C81A expression plasmids. Representative figures were generated with data from at least three independent experiments. The data are presented as the mean ± SD values. *P < 0.05 by Student's t test.

Considering the expression pattern of AGR2 in tissues, we sought to determine whether the changes in plasma lipid levels could be ascribed to the regulatory effect of AGR2 on hepatic lipid metabolism. Lipid (Oil Red O) staining of liver samples showed that lipid deposition was prominently decreased in *Agr2*^-/-^ mice compared to the WT mice fed a NCD (Figure 2A). Importantly, hepatic lipid accumulation induced by HFD feeding was dramatically alleviated in *Agr2*^-/-^ mice, whereas extensive lipid storage was observed in WT mice (Figure 2A). Furthermore, the hepatic TG and TC levels in *Agr2*^-/-^ mice were lower than those in WT mice, and this difference extended to mice fed a HFD (Figure 2B). By contrast, extensive accumulation of hepatic lipids and the increased TG/TC levels were observed in *Agr2*/Tg mice challenged with HFD feeding (Figure 2C, 2D). However, under NCD feeding conditions, hepatic lipid accumulation in *Agr2*/Tg mice was indistinguishable from that in WT mice (Figure 2C, 2D).

We further validated the effect of AGR2 on lipid metabolism using mouse primary cells. The results indicated that hepatic cells from *Agr2*^-/-^ mice showed impaired lipid droplet (LD) formation and reduced TG/TC levels in response to stearic acid (SA) compared with the primary liver cells from the WT mice (Figure 2E). Whereas the fluorescent activity of LDs and TG/TC levels were significantly increased in *Agr2*/Tg primary liver cells exposed to SA than that in WT mice (Figure 2F). Additionally, AGR2 downregulation led to a significant decrease in lipid accumulation and TG/TC levels in hepatocellular carcinoma Huh7 cells (Figure 2G, 2H). While ectopic expression of AGR2 but not the expression of the dominant-negative AGR2 mutant (AGR2-C81A, PDI motif mutation) greatly increased TG/TC levels and lipid accumulation (Figure 2I, 2J), highlighting the importance of the PDI (amino acid residues 81-84, CPHS) activity of AGR2 in the regulation of lipid metabolism. Thus, AGR2 is critical in the regulation of hepatic lipid synthesis and deposition that may depend on its PDI motif.

### AGR2 affects fatty acid uptake and utilization

**Figure 3:**
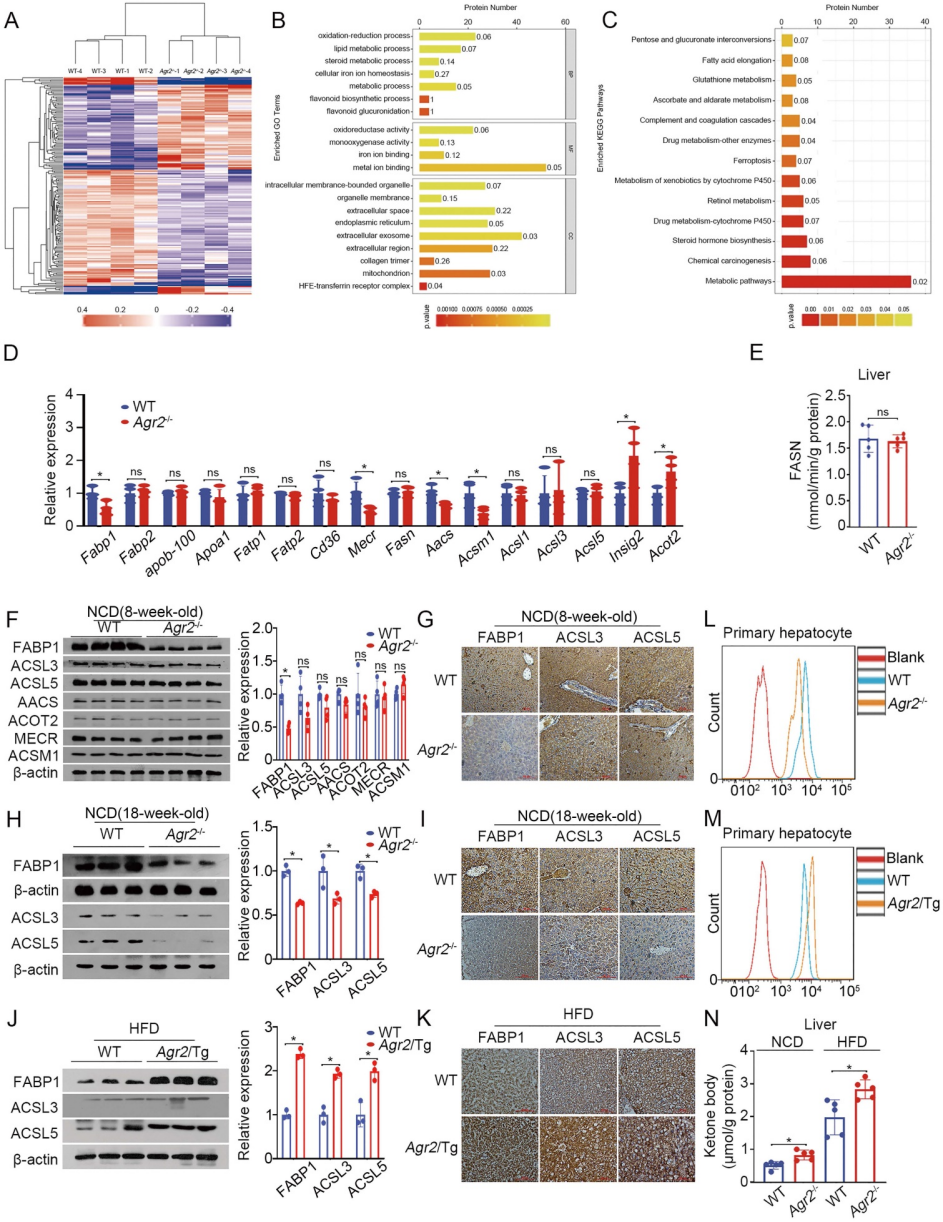
**AGR2 affects fatty acid uptake and utilization. A.** Hierarchical clustering heatmaps of differentially expressed proteins between the livers of WT and *Agr2^-/-^* mice. **B.** Results of GO term analysis of differentially expressed proteins between the livers of WT and *Agr2^-/-^* mice. **C.** Statistical results of analysis of the KEGG pathways associated with the differentially expressed proteins between the livers of WT and *Agr2^-/-^* mice. **D.** Lipid metabolism gene expression in the livers of the 8-week-old WT and *Agr2^-/-^* mice fed a NCD (n=3). **E.** Analysis of FASN activity in the cytoplasm in the livers of the 8-week-old WT and *Agr2^-/-^* mice fed a NCD (n=5). **F.** Western blot analysis of lipid metabolism protein levels in the livers of the 8-week-old WT and *Agr2^-/-^* mice fed a NCD. Quantification was performed by normalizing proteins to β-actin. **G.** Immunohistochemical staining of FABP1, ACSL3 and ACSL5 in the livers of the 8-week-old WT and *Agr2^-/-^* mice fed a NCD. **H.** Western blot analysis of FABP1, ACSL3 and ACSL5 in the livers of the 18-week-old WT and *Agr2^-/-^* mice fed a NCD. Quantification was performed by normalizing proteins to β-actin. **I.** Immunohistochemical staining of FABP1, ACSL3 and ACSL5 in the livers of the 18-week-old WT and *Agr2^-/-^* mice fed a NCD.** J.** Western blot analysis of FABP1, ACSL3 and ACSL5 in the livers of 8-week-old WT and *Agr2*/Tg mice fed a HFD for an additional 10 weeks. Quantification was performed by normalizing proteins to β-actin. **K.** Immunohistochemical staining of FABP1, ACSL3 and ACSL5 in the livers of the 8-week-old WT and *Agr2*/Tg mice fed a HFD for an additional 10 weeks. **L.** Flow cytometric analysis of lipid absorption in primary hepatocytes from WT and *Agr2^-/-^* mice. **M.** Flow cytometric analysis of lipid absorption in primary hepatocytes from WT and *Agr2*/Tg mice. **N.** Analysis of the ketone body content in the cytoplasm in the livers of 8-week-old WT and *Agr2^-/-^* mice fed a NCD or a HFD for an additional 10 weeks (n=5). Representative figures were generated with data from at least three independent experiments. The data are presented as the mean ± SD values. *P < 0.05 by Student's t test.

To explore the molecular basis for understanding AGR2-mediated regulation in lipid mechanism, we performed tandem mass tag (TMT)-based quantitative proteomics to analyze protein changes in the livers of* Agr2*^-/-^ mice at 8 weeks of age. This proteomic analysis resulted in the identification of 5196 proteins across 8 samples, four from *Agr2*^-/-^ mice and the others from WT mice. Unsupervised hierarchical clustering with Spearman correlation revealed a clear difference in the hepatic protein profiles of *Agr2*^-/-^ mice and WT mice (Figure 3A). Further analysis led to the identification of 179 proteins with a more than two-fold difference in expression (P< 0.05) upon AGR2 knockout (Figure S3A). GO classification and KEGG pathway analysis of differentially expressed proteins revealed the highest enrichment in pathways such as oxidation-reduction process, lipid metabolic process, and steroid metabolic process (Figure 3B, 3C). Among these differentially expressed proteins, 36 proteins were involved in the top metabolic pathway (Figure 3C). We performed the analysis to identify proteins that are essential for the production of these AGR2-mediated effects. Compared to those from WT mice, the samples from the livers of *Agr2*^-/-^ mice were characterized by reduced expression of several genes associated with fatty acid (FA) metabolism (Table 1 and Figure 3D). For example, the expression of FABP1, a liver-specific FABP important in FA uptake and lipid accumulation, was significantly decreased in response to AGR2 knockout. The expression of mitochondrial enoyl-acyl-carrier-protein reductase (Mecr) 23, which catalyzes type II LCFA synthesis in mitochondria, and acetoacetyl-CoA synthetase (AACS), a ketone body-utilizing enzyme responsible for cholesterol and FA synthesis in the cytosol, was reduced as well 24 (Table 1 and Figure 3D). The expression of insulin-induced gene 2 (Insig2), an ER membrane protein that negatively controls TG and TC biosynthesis 25, was markedly enhanced upon AGR2 depletion (Table 1 and Figure 3D). Notably, both the expression and enzymatic activity of fatty acid synthase (FASN), the rate-limiting enzyme in *de novo* FA synthesis, remained unchanged in hepatic samples from *Agr2*^-/-^ mice (Figure 3D, 3E). These findings suggest that protein/enzymes associated with LCFAs metabolism may be potential effectors of AGR2 knockout.

Therefore, we examined changes in proteins potentially related to FA metabolism. The results indicated that FABP1 expression was predominantly decreased in hepatic tissues of *Agr2*^-/-^ mice compared to WT mice either at 8 weeks or at 18 weeks (Figure 3F-I). Activation of LCFAs by long-chain acyl-CoA synthetases (ACSLs) is essential for their utilization in cells 26. Both ACSL3 and ACSL5 were slightly altered upon loss of AGR2 at 8 weeks of age in mice (Figure 3F, 3G), however, they significantly declined in liver sections from *Agr2*^-/-^ mice at 18 weeks (Figure 3H, 3I), implicating that loss of AGR2 down-regulated proteins associated with FA uptake and activation with age. Moreover, an increase in FABP1 and ACSL5 was clearly present in liver samples from *Agr2*/Tg mice compared to those from WT mice fed with HFD, while ACSL3 expression was marginally altered (Figure 3J, 3K), supporting the observations that AGR2 exhibited an effect on FA metabolism. We next determined whether AGR2 was associated with alterations in FA uptake. Primary hepatic cells from either *Agr2*^-/-^ or *Agr2*/Tg mice were treated with the fluorescent LCFA analog boron-dipyrromethene (BODIPY), fluorescence activity in cells was measured by flow cytometry. The results demonstrated that reduced fluorescence was obviously observed in hepatic cells from *Agr2*^-/-^ mice than that in WT mice (Figure 3L), whereas LCFA uptake was evidenced in cells from the liver of *Agr2*/Tg mice (Figure 3M). Moreover, the ketone body content was markedly increased in hepatic tissues of *Agr2*^-/-^ mice fed either a NCD or a HFD (Figure 3N), supporting the idea that AGR2 knockout facilitates FA oxidation. In contrast, accumulation of acetyl-CoA in both the cytoplasm and mitochondria was evidenced upon AGR2 overexpression in HepG2 cells, and reduced ketone body production was observed in the livers of *Agr2*/Tg mice (Figure S3B, S3C), confirming the function of AGR2 in FA metabolism.

Taken together, AGR2 deficiency leads to suppression of FA uptake and activation, which might be largely dependent on FABP1.

### AGR2-mediated effect on fatty acid metabolism depends on FABP1

**Figure 4:**
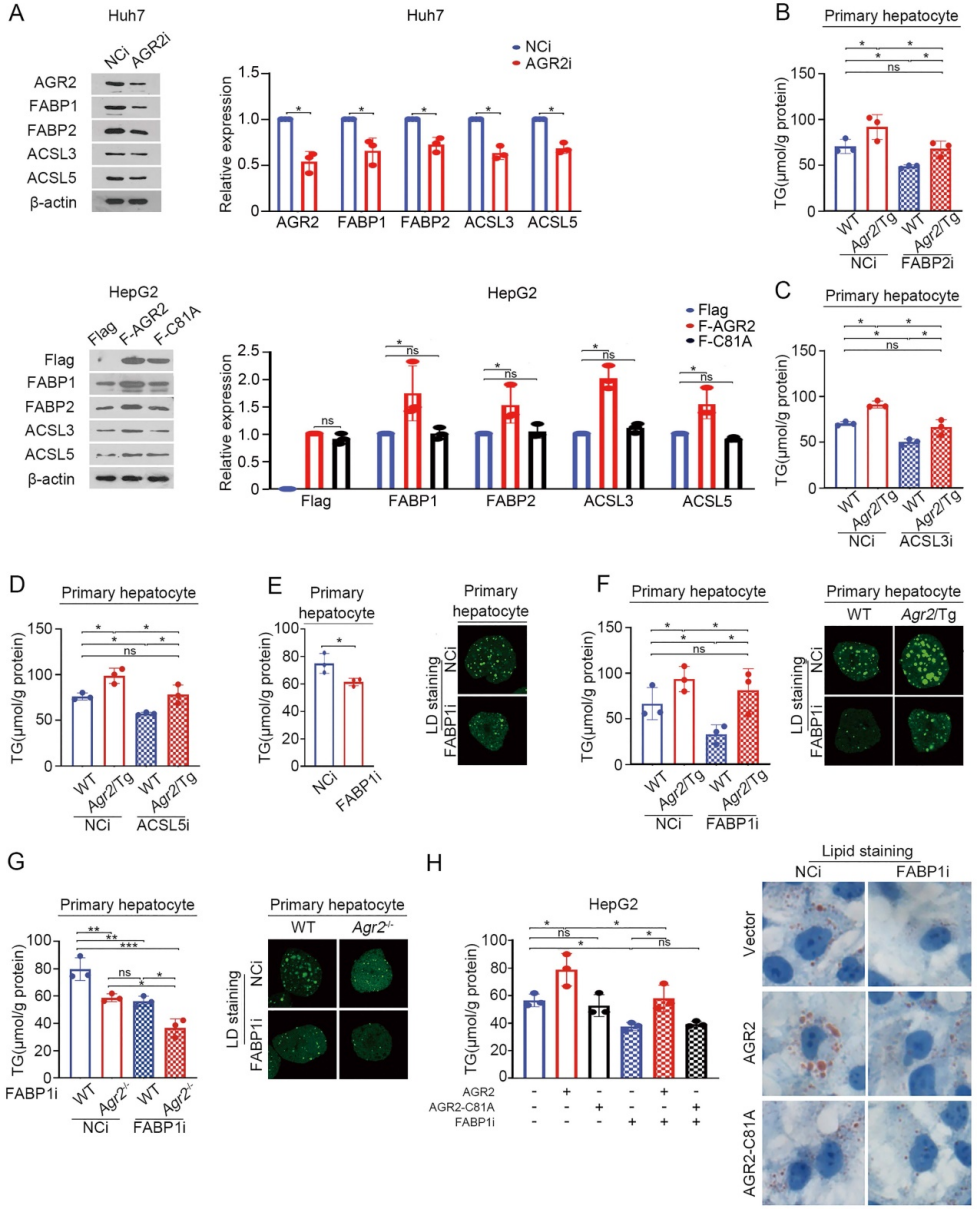
** AGR2-mediated effect on fatty acid metabolism depends on FABP1. A.** Western blot analysis of lipid metabolism protein levels in whole-cell lysates from Huh7 cells treated with siRNA targeting AGR2. Western blot analysis of lipid metabolism protein levels in whole-cell lysates from HepG2 cells treated with AGR2 and AGR2-C81A expression plasmids. Quantification was performed by normalizing proteins to β-actin. **B.** TG in primary hepatocytes from WT and *Agr2*/Tg mice treated with siRNA targeting FABP2. **C.** TG in primary hepatocytes from WT and *Agr2*/Tg mice treated with siRNA targeting ACSL3. **D.** TG in primary hepatocytes from WT and *Agr2*/Tg mice treated with siRNA targeting ACSL5. **E.** TG and lipid droplet staining in primary hepatocytes from WT mice treated with siRNA targeting FABP1. **F.** TG and lipid droplet staining in primary hepatocytes from WT and *Agr2*/Tg mice treated with siRNA targeting FABP1. **G.** TG and lipid droplet staining in primary hepatocytes from WT and *Agr2*^-/-^ mice treated with siRNA targeting FABP1. **H.** TG content in HepG2 cells treated with AGR2 and AGR2-C81A expression plasmids and siRNA targeting FABP1. Lipid staining in HepG2 cells treated with AGR2 and AGR2-C81A expression plasmids and siRNA targeting FABP1. Representative figures were generated with data from at least three independent experiments. The data are presented as the mean ± SD values. *P < 0.05, **P < 0.01, ***P < 0.001 by Student's t test.

We next determine whether FABPs and ACSLs are sufficient effector(s) for AGR2 mediated activity on FA metabolism. AGR2 deficiency suppressed the expression of FABP1and FABP2, while ACSL3 and ACSL5 slightly affected in Huh7 cells (Figure 4A). Overexpression of AGR2 increased the abundance of FABP1 and FABP2, while had a moderate effect on ACSL3 and ACSL5 (Figure 4A). Notably, the expression of FABP1, FABP2, ACSL3, and ACSL5 almost remained unchanged in cells overexpressing the enzymatically inactive AGR2 (AGR2-C81A), indicating that the PDI motif in AGR2 is critical for regulation of these candidate proteins. TG production analysis was used to evaluate the involvement of these four proteins. The results showed that FABP1 depletion exhibited more potent suppression on TG synthesis than that of FABP2, ACSL3, and ACSL5, when AGR2 level was high in cells (Figure 4B-F), highlighting the importance of FABP1 in the delivery effect of AGR2. Given the significant response to AGR2, FABP1 is selected as a model for further investigation. As is shown in Figure 4E, FABP1 knockdown significantly inhibited TG content and lipid-drop (LD) formation in primary hepatic cells. An increase in TG synthesis and LD formation were shown in liver cells from *Agr2*/Tg mice, but this enhancement was noticeably alleviated in the absence of FABP1 (Figure 4F). Moreover, FABP1 depletion exacerbated the suppressive effect of AGR2 deficiency on TG content and LD formation (Figure 4G). We also found that dominant-negative AGR2 (AGR2-C81A) lost its impact on FABP1 and TG accumulation (Figure 4H). Therefore, FABP1 plays an important role in the AGR2-mediated effect on lipid accumulation.

### AGR2 acts as a stabilizer for FABP1

**Figure 5:**
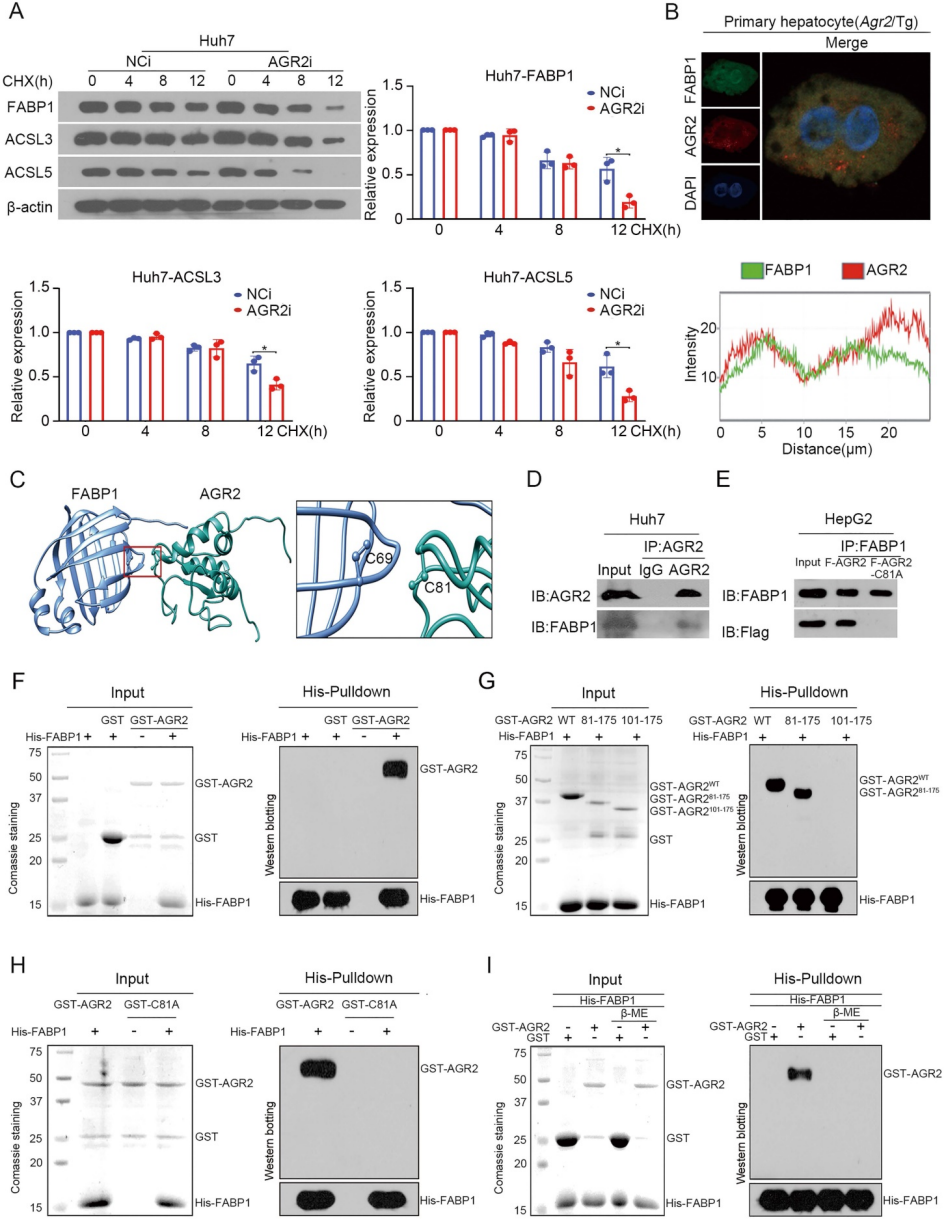
** AGR2 acts as a stabilizer for FABP1. A.** Huh7 cells were treated with 10 µM CHX for 0, 4, 8 and 12 h. FABP1, ACSL3 and ACSL5 were detected by western blotting. Quantification was performed by normalizing proteins to β-actin. **B.** Immunofluorescence staining of FABP1 and AGR2 in primary hepatocytes from *Agr2*/Tg mice. **C.** *In silico* prediction of interactions between AGR2 and FABP1. Green, AGR2; blue, FABP1. **D.** Co-immunoprecipitation analysis of the interaction between AGR2 and FABP1 using Huh7 cell lysates.** E.** Co-immunoprecipitation analysis of the interaction between AGR2 and FABP1 using cell lysates in HepG2 cells treated with AGR2 and AGR2-C81A expression plasmids. **F.** His pulldown analysis of the interaction between AGR2 and FABP1.** G.** His pulldown assays show that the mutation of thioredoxin motif blocks the AGR2-FABP1 interaction. **H.** His pulldown assays showing the direct interaction of AGR2 and its deletion mutants with FABP1.** I.** His pulldown assays show that β-ME blocks the AGR2-FABP1 interaction. Representative figures were generated with data from at least three independent experiments. The data are presented as the mean ± SD values. *P < 0.05 by Student's t test.

We next explored mechanistic insight into the regulation of AGR2 on FABP1. Endogenous FABP1 gradually declined over time in response to cycloheximide (CHX), a protein synthesis inhibitor, while AGR2 depletion resulted in significant decreases in the half-life of FABP1 (Figure 5A). Also, the ACSL3 and ACSL5 proteins, particularly ACSL5, exhibited instability in response to low levels of AGR2 (Figure 5A). These results suggest that AGR2 may act as a chaperone to stabilize substrate proteins.

We focused our attention to examine whether the FABP1 stability was ascribed to the interactions with AGR2. Immunofluorescence staining clearly showed that AGR2 and FABP1 were colocalized in the cytosol of primary liver cells from *Agr2*/Tg mice (Figure 5B). The docking results *in silico* revealed that Cys81 of AGR2 might be required for binding to Cys69 in FABP1 (Figure 5C). Coimmunoprecipitation results clearly revealed that FABP1 was efficiently immunoprecipitated in immunocomplexes with an antibody specific for AGR2 in Huh7 cells (Figure 5D). Reciprocally, AGR2 has readily detected in immunocomplexes precipitated with an anti-FABP1 antibody, but a mutation in Cys81 of AGR2 (AGR2-C81A) was unable to precipitate FABP1 in cells (Figure 5E), supporting the involvement of the Cys81 in PDI motif in the formation of the AGR2/FABP1 complex. Furthermore, purified recombinant FABP1 protein (rFABP1) (Figure S3D), recombinant full-length AGR2 (rAGR2), and AGR2 truncation mutant (Figure S3E) proteins were employed to determine the interaction region(s) of these two proteins. Histidine (His) pulldown assays revealed that the GST-AGR2 fusion protein but not GST specifically coprecipitated with the His-FABP1 protein (Figure 5F). Moreover, rFABP1 interacted with both rAGR2 and the AGR2 truncation mutant containing PDI and KTEL motifs (amino acid residues 81-175, AGR2^81-175^) (Figure 5G), however, it failed to bind the AGR2 mutant (amino acid residues 101-175, AGR2^101-175^) lacking the PDI motif (amino acid residues 81-84) or the AGR2-C81A mutant (Figure 5H), providing fundamental evidence that AGR2 directly interacts with FABP1 via the Cys 81 in PDI motif. Since AGR2 exerts isomerase and chaperone activity, the formation of disulfide bonds between AGR2 and FABP1 was then investigated in the presence of the reducing agent β-mercaptoethanol (β-ME). The pulldown assay results showed that the physical interaction between AGR2 and FABP1 was disrupted by β-ME (Figure 5I), highlighting the importance of the disulfide bond in AGR2-mediating substrate stability. Additionally, ACSL5 appeared to be a substrate and an effector of AGR2 because AGR2 could bind to ACSL5 (Figure S3F) and AGR2-mediated lipid synthesis was partially reduced when ACSL5 was downregulated in cells (Figure S3G). These results provide substantial evidence that effector proteins, particularly hepatic FABP1, are substrates of AGR2 and mediate the regulatory effect of AGR2 on lipid synthesis.

### AGR2 influences FA absorption in the intestine

**Figure 6:**
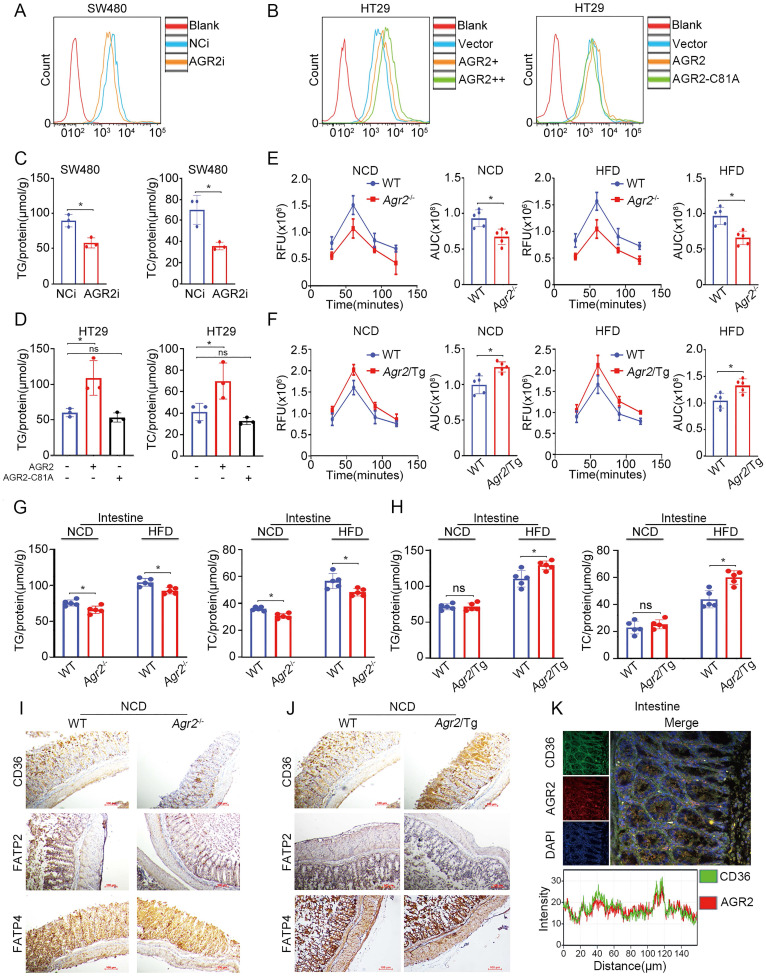
** AGR2 influences FA absorption in the intestine.** The 8-week-old mice were fed a NCD or a HFD for an additional 10 weeks. **A.** Flow cytometric analysis of lipid absorption in SW480 cells treated with siRNA targeting AGR2. **B.** Flow cytometric analysis of lipid absorption in HT29 cells treated with AGR2 and AGR2-C81A expression plasmids. **C.** TG and TC contents in SW480 cells treated with siRNA targeting AGR2. **D.** TG and TC contents in HT29 cells treated with AGR2 and AGR2-C81A expression plasmids. **E.** Lipid absorption in WT and *Agr2^-/-^* mice fed a NCD or a HFD (n=5). **F.** Lipid absorption in WT and *Agr2*/Tg mice fed a NCD or a HFD (n=5). **G.** TG and TC contents in the intestines of WT and *Agr2^-/-^* mice fed a NCD or a HFD (n=5). **H.** TG and TC contents in the intestines of WT and *Agr2*/Tg mice fed a NCD or a HFD (n=5). **I.** Immunohistochemical staining of FA uptake proteins in the intestines of WT and *Agr2^-/-^* mice fed a NCD.** J.** Immunohistochemical staining of FA uptake proteins in the intestines of WT and *Agr2*/Tg mice fed a NCD. **K.** Immunofluorescence staining of CD36 and AGR2 in the intestines. Representative figures were generated with data from at least three independent experiments. The data are presented as the mean ± SD values. *P < 0.05 by Student's t test.

AGR2 is highly expressed in intestinal enterocytes, where hydrolysis of dietary TGs generates FAs, which are then taken up by absorptive enterocytes and serve as important contributors to TG resynthesis. We then sought to examine whether FA absorption is perturbed after AGR2 knockout. Indeed, SW480 cells with AGR2 depletion exhibited decreased uptake of fluorescently labeled FAs (Figure 6A), whereas HT29 cells with ectopic expression of AGR2, but not the AGR2-C81A mutant, exhibited a dose-dependent increase in FA uptake compared to controls (Figure 6B and Figure S4A). Accordingly, reduced TG/TC content and lipid staining were observed in AGR2-silenced SW480 cells, while lipid accumulation occurred upon AGR2 overexpression in HT29 cells (Figure 6C, 6D and Figure S4B, S4C), suggesting that AGR2 has a positive impact on FA uptake. This effect was further evaluated in mice with genetic manipulation of AGR2. As shown in Figure 6E, intestinal absorption of FAs, as determined by the fluorescent activity of BODIPY 20, was significantly impaired in *Agr2*^-/-^ mice compared with their WT littermates under NCD feeding conditions, and this difference was more pronounced in *Agr2*^-/-^ mice fed a HFD. Under both NCD and HFD feeding conditions, the FA absorption capacity was obviously enhanced in *Agr2*/Tg mice compared with WT mice (Figure 6F). Additionally, the pattern of changes in the intestinal TG/TC contents was similar to those observed in hepatic tissue samples from *Agr2*^-/-^ and *Agr2*/Tg mice fed either a NCD or a HFD (Figure 6G, 6H). These results clearly demonstrated a role of AGR2 in exogenous LCFA absorption and lipid resynthesis in the intestine.

Since a cluster of differentiation 36 (CD36), fatty acid transport protein 2 (FATP2), and FATP4 are membrane receptors involved in intestinal FA uptake and lipid metabolism 27, it was necessary to investigate whether AGR2 regulates these membrane proteins. Compared with control mice, *Agr2*^-/-^ mice fed a NCD exhibited more decreased CD36 expression than that in FATP2 and FATP4 expression (Figure 6I). Again, we observed a high level of CD36 but not FATP2 or FATP2 in *Agr2*/Tg mice on a NCD (Figure 6J). The immunofluorescence staining results indicated that AGR2 was highly colocalized with CD36 in the intestine, suggesting that AGR2 and CD36 may cooperate to mediate FA influx (Figure 6K). In intestinal enterocytes, both liver (FABP1) and intestinal (FABP2) FABPs are involved in the uptake and trafficking of lipids 28. Similar to the observations in hepatic samples, FABP2 was found to be a substrate of AGR2 in the intestine, because it was regulated by and bound to AGR2 (Figure S4D-H). Thus, AGR2 deficiency impairs LCFA absorption and lipid accumulation and at least partially accounts for the destabilization of intestinal CD36, FABP1, and FABP2.

## Discussion

In the present study, we provided evidence demonstrating a novel function of AGR2 in the regulation of lipid metabolism. AGR2 deficiency displayed a reduction in adiposity that was ascribed to impaired hepatic LCFA uptake/absorption and activation in the liver and intestine, which was associated with decreased plasma lipid levels and resistance to HFD-induced lipid accumulation. However, AGR2 overexpression as indicated by *Agr2*/Tg mice showed an increase in body fat and the levels of lipid parameters under HFD but not NCD feeding conditions. We also identified novel interacting proteins of AGR2, including FABP1, FABP2, and ACSL5, and particularly demonstrated that AGR2-dependent chaperone activity is important for the stability of FABP1.

It has been reported that the PDI family member ERp44, ERdj5, and PDIA1 contribute to the efficient folding of adiponectin, LDL receptor, and apoB100. Our study first places AGR2 in the regulatory pathway of lipid metabolism. AGR2 is expressed in the liver and digestive tract rather than in adipose and muscle tissues. Screening of differentially expressed proteins in hepatic samples from AGR2-deficient mice by proteomics revealed alterations in FA uptake/activation and cholesterol metabolism. Further analysis led us to identify FABP1 as a novel substrate of AGR2, mediating the effect of AGR2 on FA uptake and activation. FABP1 is an abundantly expressed protein that binds LCFAs and acts as an intracellular FA transporter in hepatocytes and the small intestinal mucosa 29, 30. We demonstrated that AGR2 directly interacted with FABP1, depending on the Cys81 residue in the PDI motif, highlighting the importance of the isomerase and/or chaperone activity of AGR2 in substrate folding and stability. The FABP1 protein contains a Cys residue, docking analysis indicates that Cys69 in FABP1 may be important in the formation of a disulfide bond with Cys81 in AGR2. Further investigation is required to the definition of the function of Cys69 in FABP1 folding and stability. Like other chaperone/isomerase of the PDI family 31, AGR2 also exerts its functions via diverse substrates or client proteins 32. In addition to FABP1, FABP2 and ACSL5 was found to interact with AGR2, which might facilitate FA acylation and activation, enhancing the effect of AGR2 on FA metabolism. Previous studies have demonstrated that interactions between AGR2 and mucin-2 via the Cys residue to form mixed disulfide bonds are involved in mucin processing 22. Guo et al and our study have defined the interactions between AGR2 and vascular endothelial growth factor A (VEGFA) through the formation of a disulfide bond, which results in the enhancement of VEGF/VEGFR2 signaling to promote tumor angiogenesis 33, 34. Clearly, the functions of AGR2 are dependent mainly on its substrate/partner proteins under pathophysiological conditions. The single Cys81 residue in AGR2 is critical in its substrate binding activity.

AGR2 is a novel regulator in FA uptake/absorption and activation in the liver and intestine. The contribution of AGR2 to lipid metabolism could be explored to develop a potential target for lipid-lowering therapy.

## Supplementary Material

Supplementary figures.

## Figures and Tables

**Table 1:** Differentially expressed proteins of lipid metabolism between the livers of WT and *Agr2^-/-^* mice

Protein Name	Fold Change
AACS	0.7742037
GPX1	0.7795029
SRD5A1	0.7880489
FDX1	0.8087367
HPGD	0.8268217
FABP1	0.8300376
MECR	0.8424256
ACSM1	0.8474276
PLTP	0.8515036
ACLY	0.8698593
LDLRAP1	1.232275
CYP39A1	1.2400817
ACOT2	1.2461947
BSCL2	1.3825145
INSIG2	1.4901663

## Abbreviations

AGR2: Anterior gradient 2
FABP1: fatty acid binding protein-1
HDL-C: high-density lipoprotein cholesterol
HFD: high-fat diet
LDL-C: low-density lipoprotein cholesterol
NCD: normal chow diet
PDI: protein disulfide isomerase
TC: total cholesterol
TG: triglyceride
