# Supplementary material for: Anterior gradient 2 increases long-chain fatty acid uptake via stabilizing FABP1 and facilitates lipid accumulation
Source: Int J Biol Sci. 2021 Feb 8;17(3):834–47. doi: 10.7150/ijbs.57099 (PMC7975708; doi:10.7150/ijbs.57099)

Anterior Gradient 2 Increases Long-chain Fatty Acid Uptake via Stabilizing FABP1 and Facilitates Lipid Accumulation

Yunqiu Wang[1,2], Mengqi Jia[1], Chuanjie Liang[1,2], Nan Sheng[1], Xiaodan Wang[1], Fang Wang[1], Yanhai Luo[1,2], Jin Jiang[1], Liangyu Cai[1], Huanmin Niu[1], Deyu Zhu[2], Effat Un Nesa[1], Charles YF Young[3], Huiqing Yuan[1,2]*

Supplementary Materials and Methods

Cell lines and cell culture

The human liver cancer lines (HepG2 and Huh-7), the human colon cancer cell line SW480 and HEK293T cells were maintained in DMEM medium (Hyclone, Logan, USA). The human colon cell line HT29 was maintained in McCoys 5A medium (Hyclone, Logan, USA). All cells were purchased from the Cell Bank of Chinese Academy of Sciences (Shanghai). All cells were supplemented with10% fetal bovine serum (Gibco, Grand Island, NY), 100 U/mL of penicillin and 100 l g/mL of streptomycin, at 37 °C in a humid atmosphere (5% CO_2 –95% air). All cells were harvested by brief incubation in 0.25% (w/v) EDTA-PBS.

Transient transfection of plasmids and siRNAs

The cells were transiently transfected with pcDNA3.1, pcDNA3.1-AGR2 and pcDNA3.1-AGR2-C81A for 48 h using Lipofectamine 2000 (Invitrogen Life Technologies, Carlsbad, CA, USA) according to the manufacturer's instruction. For the siRNA assay, AGR2 siRNA, ACSL5 siRNA, FABP1 siRNA, FABP2 siRNA, mouse

AGR2 siRNA, mouse FABP1 siRNA, mouse FABP2 siRNA, mouse ACSL3 siRNA and mouse ACSL5 siRNA (GenePharma, Shanghai, China) were transfected into the cells for 48 h. Scramble siRNA served as a control. After transfection, the cells were subjected to treatment. At least three independent experiments were performed. The siRNA sequences were listed in Supplementary Table S1.

Western blotting assay

Western blotting assay was performed to analyze the expressions of proteins in cells. Cells were washed with ice-cold PBS and cell lysates were prepared using SDS buffer containing fresh protease inhibitor mixture (50 μg/mL aprotinin, 0.5 mM phenylmethanesulfonyl fluoride (PMSF), 1 mM sodium orthovanadate, 10 mM sodium fluoride and 10 mM bglycerolphosphate). Proteins were quantified using the BCA protein assay. Samples containing equal amounts of protein (40 μg protein per lane) from lysates were separated by 10–12% SDS–PAGE and electrophoretically transferred onto polyvinylidene fluoride (PVDF) membranes. After being blocked with 5% fat-free dry milk in TBS (20 mM Tris–HCl (pH 7.6), 150 mM NaCl) for 1 h at room temperature, the membranes were immunoblotted with primary antibodies overnight at 4 °C, followed by incubation with HRP-conjugated secondary antibodies for 1 h at room temperature. Membranes were visualized by enhanced chemiluminescence detection system (Millipore) and followed by exposure to X-ray films. The bands in western blotting showed were densitometrically quantified with ImageJ software, and normalized by internal controls.

Real-time reverse-transcription PCR (RT-PCR)

Total RNA was extracted using a Trizol Kit (Takara, Dalian, China). cDNA was prepared using a PrimerScript RT reagent Kit with gDNA Eraser (Takara, Dalian, China) according to the manufacturer's protocol. Real-time PCR was performed with SYBR Green (Takara, Dalian, China). The cycling conditions for: 94 °C for 2 min and 30 s; followed by 40 cycles of: 94 °C for 35 s; 60 °Cfor 35 s; 72 °C for 30 s. Changes in the transcript level were calculated using the DD Ct method. The expressions of genes were normalized against that of a housekeeping gene and plotted as relative change in the expression with respect to control. Each reaction was performed in triplicate. The primers of the target genes are listed in supplementary Table S2.

Hematoxylin and eosin staining and immunohistochemical analysis

The tissue specimens were deparaffinized and rehydrated. The tissue specimens were sectioned at a 5- l m thickness, and the specimens were prepared for hematoxylin and eosin (H&E) staining and IHC analysis. The H&E staining was performed by routine methods. The appropriate primary antibodies were then added onto the sections and incubated overnight at 4 °C. After being stained with IgG conjugated HRP and DAB (Vector Laboratories), samples were counterstained with hematoxylin and subjected to capture images by microscopy (Nikon).

Oil Red O staining

Oil Red O is a lysochrome diazo dye (fat-soluble) used for staining neutral TGs and lipids. Cells and tissue specimens were fixed with 10% formaldehyde at room temperature for 10 min, the 10% formaldehyde was discarded, and fresh 10% formaldehyde was added for 1 h. The samples were washed sequentially twice with

water and once with 60% isopropanol after fixation. The cells and tissue specimens were stained with Oil Red O working solution for 30 min at room temperature and washed five times with water to remove nonspecifically bound dye. Micrographs were then acquired.

Immunofluorescence staining

Cells were grown on cover slips, fixed with ice-cold methanol/acetone (1:1) and incubated with 3% bovine serum albumin in phosphate-buffered saline with 0.1% Triton X-100 for 20 min. After washing, cells were incubated with primary antibodies prior to rinsed with phosphate-buffered saline, then immunostained with secondary antibodies and nuclei were stained with DAPI (Sigma-Aldrich, USA). Fluorescence images were captured using a confocal microscopy (Carl Zeiss, Germany).

Flow cytometry for Lipid absorption

Treated cells were incubated with C1-Bodi Fluro 500/510-C12. Control cells were not incubated with. After 10 min of incubation cells were washed once with PBS, then trypsinized and centrifuged for 5 min at 800g. Cells were washed twice more with PBS and resuspended in 0.4 ml of PBS for flow cytometry to measure lipid uptake.

LDs staining

Cells were grown on cover slips, followed by fixation with 4% PFA diluted in PBS for 15 min at RT. Cells were washed with PBS three times and permeabilized with 0.2% Triton X-100 diluted in PBS for 10 min. LDs were stained with either 1 μg/ml of BODIPY 493/503 (Invitrogen) in PBS for 10 min at RT. Fluorescence images were captured using a confocal microscopy (Carl Zeiss, Germany).

Purification of His-AGR2 and GST-AGR2 fusion protein

The Pet15b-AGR2 and pGEX-6p-1-AGR2 were transformed into E. coli strain BL21 DE3. The transformants were grown at 37°C in LB medium (100 μg/mL Ampicillin) and induced with 0.2 mM isopropyl-β-D-1-thiogalactopyranoside (IPTG) for 12h at 18°C. The harvested cells were resuspended in Tris buffer (20 mM Tris-HCl, 150mM NaCl, pH 8.0), and disrupted by sonication on ice. Co-NTA column and GST-Sifenose column (Sangon Biotech, Shanghai, China) was used to extract the His-AGR2 and GST-AGR2 fusion protein from the supernatant. His-AGR2fusion protein follows by S-Sepharose FF column for further purification. GST-AGR2 fusion protein was eluted by elution buffer (20 mM Tris-HCl, 150mM NaCl, 25mM GSH, pH 8.0), then use dialysis (20 mM Tris-HCl, 150mM NaCl, pH 8.0) to remove GSH. His and GST tag was located on N-terminal of AGR2.

Purification of His-FABP1 fusion protein

The Pet28a-FABP1 was transformed into E. coli strain BL21 DE3. The transformants were grown at 37°C in LB medium (40 μg/mL Kanamycin) and induced with 0.2 mM isopropyl-β-D-1-thiogalactopyranoside (IPTG) for 12h at 18°C. The harvested cells were resuspended in Tris buffer (20 mM Tris-HCl, 150mM NaCl, pH 8.0), and disrupted by sonication on ice. Co-NTA column was used to extract the His-FABP1 fusion protein from the supernatant. His-FABP1 fusion protein was eluted by elution buffer (20 mM Tris-HCl, 150mM NaCl, 250mM Imidazole, pH 8.0), then use dialysis (20 mM Tris-HCl, 150mM NaCl, pH 8.0) to remove Imidazole.

Supplemental Tables

Supplemental Table 1. siRNA sequences RT-PCR Primer

Target gene	Primers
Scramble siRNA	Sense 5'-UUC UCC GAA CGU GUC ACG UTT-3'
	Anti-sense 5'-ACG UGA CAC GUU CGG AGAATT-3'
Human AGR2 siRNA	Sense 5'- UUGUCAAGCAACAGAGCUGUAUCUG-3'
	Anti-sense 5'-CAGAUACAGCUCUGUUGCUUGACAA-3'
Human FABP1 siRNA	Sense 5'-GUUGGAAGGUGACAAUAAACU-3'
	Anti-sense 5'-UUUAUUGUCACCUUCCAACUG-3'
Human ACSL5 siRNA	Sense 5'-GCACCAGAGAAGAUAGAAATT-3'
	Anti-sense 5'-UUUCUAUCUUCUCUGGUGCTT-3'
Mouse ACSL3 siRNA	Sense 5'-CAGCUCUGUUGUACGACAACA-3'
	Anti-sense 5'-UUGUCGUACAACAGAGCUGUG-3'
Mouse ACSL5 siRNA	Sense 5'- CGAGGACCUCAUCCAGAAAGG-3'
	Anti-sense 5'- UUUCUGGAUGAGGUCCUCGGG-3'
Mouse FABP1 siRNA	Sense 5'-GCUGGAAGGUGACAAUAAAUU-3'
	Anti-sense 5'-UUUAUUGUCACCUUCCAGCUU-3'
Mouse FABP2 siRNA	Sense 5'-GAGAAUUUAGACAAGGCUAUA -3'
	Anti-sense 5'-UAGCCUUGUCUAAAUUCUCUU -3'

Supplementary Table 2. qPCR Primer

Target gene	Primers

Mouse *Acsl1*	F: 5'-TGCCAGAGCTGATTGACATTC-3'
	R: 5'-GGCATACCAGAAGGTGGTGAG-3'
Mouse *Acsl3*	F: 5'-GGAGAGTTTGACCCTGACGG-3'
	R: 5'-GCTGCCTCTAACTTTCCCGAG-3'
Mouse *Acsl5*	F: 5'-GTACCTGGGCTCCTGTCTTT-3'
	R: 5'-TTCTGTACCCAGCGTGTACT-3'
Mouse *Accs1*	F: 5'-GCTGACATCGGTTGGATCAC-3'
	R: 5'-ACGACCAGCATCAGGGTAAA-3'
Mouse *Dhcr24*	F: 5'-GTGGAGCCCTTGGTGTCTAT-3'
	R: 5'-AACAGGCCATACTTGTGGGA-3'
Mouse *apob-100*	F: 5'-TTCGCTAAGAGCAGTGTCCA-3'
	R: 5'-GTTCTCAACCAGAGGTGGGA-3'
Mouse *Apoa1*	F: 5'-TGTGGATGCGGTCAAAGACA-3'
	R: 5'-GGAGCGCAGGATAGACYCAC-3'
Mouse *Fatp1*	F: 5'-CGCCCATGTGCTCTATGACT-3'
	R: 5'-ACACAGTCATCCCAGAAGCG-3'
Mouse *Fatp2*	F: 5'-AGCTGTGGAGGAGGTTCTCC-3'
	R: 5'-GATGATTGATGGTTGCCGCT-3'
Mouse *Fabp1*	F: 5'-AAGGGGGTGTCAGAAATCGT-3'
	R: 5'-ACTCATTGCCGGACCACTTTG-3'
Mouse *Fabp2*	F: 5'-CTGATTGCTGTCCGAGAGGT-3'
	R: 5'-AAGAATCGCTTGGCCTCAAC-3'

Mouse *Fasn*	F: 5'-CAAGTGTCCACCAACAAGCG-3'
	R: 5'-GGAGCGCAGGATAGACTCAC-3'
Mouse *Cd36*	F: 5'-GCAGCCTCCTTTCCACCTTT-3'
	R: 5'-TGTCTGGATTCTGGAGGGGT-3'
Mouse *β-actin*	F: 5'-GTGACGTTGACATCCGTAAAGA-3'
	R: 5'-GCCGGACTCATCGTACTCC-3'
Mouse *Agr2*	F: 5'-GGACTCGGCCCAAACTAC-3'
	R: 5'-GAATGACCATCAAGGGTCTGTT-3'
Mouse *Aacs*	F: 5'-ACCGTGTGGTCGGCTATCTA-3'
	R: 5'-TTTACACCAAAGTCCGGCGA-3'
Mouse *Mecr*	F: 5'-TCCGAGACAGACCCGACAT-3'
	R: 5'-ACAGTTGAGAGCCAATCGGG-3'
Mouse *Insig2*	F: 5'-TGCTGGAGGCATAACGATGG-3'
	R: 5'-TCCCAAGGCTCCCAAAGAAC-3'
Mouse *Acsm1*	F: 5'-CCAGGCGTGTATATCGAATGGT-3'
	R: 5'-ACTGCTGATCCCCGTTTCTG-3'

Supplemental Table 3. Antibody

Antibody	Company	Catalog number	Dilutions
Rabbit anti-AGR2	Proteintech	Cat# 12275-1-AP;	WB: 1:500
			IHC: 1:200
Mouse anti-AGR2	Santa	Cat#sc-101211	IF: 1:50

			IP: 0.5μg
Mouse anti-β-actin	Proteintech	Cat# 60008-1-Ig	WB: 1:500
Mouse anti-GST	Santa	Cat#sc-138	WB: 1:500
Mouse anti-Flag(M2)	Sigma	Cat#F1804	WB: 1:1000
Rabbit anti-FABP1	Proteintech	Cat#13626-1-AP	WB: 1:2000
			IHC: 1:500
			IF: 1:200
			IP: 0.5μg
Rabbit anti-ACSL3	Proteintech	Cat#20710-1-AP	WB: 1:500
			IHC: 1:200
Rabbit anti-ACSL5	Proteintech	Cat#15708-1-AP	WB: 1:500
			IHC: 1:200
Rabbit anti-AACS	Proteintech	Cat#13815-1-AP	WB: 1:200
Rabbit anti-ACOT2	Proteintech	Cat#15633-1-AP	WB: 1:500
Rabbit anti-CD36	Proteintech	Cat#18836-1-AP	IHC: 1:400
			IF: 1:200
Rabbit anti-FATP2	Proteintech	Cat#14048-1-AP	IHC: 1:200
Rabbit anti-FATP4	Proteintech	Cat#11013-1-AP	IHC: 1:200
Rabbit anti-FABP2	Affinity	Cat#DF6508	WB: 1:500
			IHC: 1:200
Rabbit anti-MECR	Abcam	Cat#ab254707	WB: 1:500
Rabbit anti-ACSM1	Abnova	Cat#PAB24493	WB: 1:500

Supplemental Figure Legends

Supplemental Figure 1. AGR2 knockout reduces serum lipid levels and fat accumulation. A. AGR2 gene expression in the tail of WT and *Agr2*$^{-/-}$ mice (n=3). **B.** Western blot analysis of AGR2 in the tail of WT and *Agr2*$^{-/-}$ mice. Quantification was performed by normalizing proteins to β-actin. **C.** Immunohistochemical staining of AGR in the liver of WT and *Agr2*$^{-/-}$ mice. **D.** Food intake of the 8-week-old/18-week-old WT and *Agr2*$^{-/-}$ mice fed a NCD (n=5). **E.** Water intake of the 8-week-old/18-week-old WT and *Agr2*$^{-/-}$ mice fed a NCD (n=5). **F.** Weights of kidney, heart, liver, spleen and lung (left) and length of tibla (right) of the 18-week-old WT and *Agr2*$^{-/-}$ mice fed a NCD (n=5). **G.** Weights of kidney, heart, liver, spleen and lung (left) and length of tibla (right) of the 8-week-old WT and *Agr2*$^{-/-}$ mice fed a HFD for an additional 10 weeks (n=5). **H.** eWAT mass and eWAT percentage of the 8-week-old WT and *Agr2*$^{-/-}$ mice fed a NCD or a HFD for an additional 10 weeks (n=5). **I.** subWAT mass and subWAT percentage of the 8-week-old WT and *Agr2*$^{-/-}$ mice fed a NCD or a HFD for an additional 10 weeks (n=5). Representative figures were generated with data from at least three independent experiments. The data are presented as the mean ± SD values. *P < 0.05, **P < 0.01, ***P < 0.001 by Student's t test.

Supplemental Figure 2. AGR2 knockout reduces serum lipid levels and fat accumulation. A. AGR2 gene expression in the tail of WT and *Agr2*/Tg mice (n=3). **B.** Elisa analysis of AGR2 in the serum of WT and *Agr2*/Tg mice (n=5). **C.** Western blot

analysis of AGR2 in the tail of WT and *Agr2*/Tg mice. Quantification was performed by normalizing proteins to β-actin. **D.** Immunohistochemical staining of AGR in the liver of WT and *Agr2*/Tg mice. **E.** Body weight of WT and *Agr2*/Tg mice during the 10 weeks of NCD feeding (n=5). **F.** Body weight, fat mass, lean body mass and fat percentage of the 18-week-old WT and *Agr2*/Tg mice fed a NCD (n=5). **G.** Weights of kidney, heart, liver, spleen and lung (left) and length of tibla (right) of the 18-week-old WT and *Agr2*/Tg mice fed a NCD (n=5). **H.** Weights of kidney, heart, liver, spleen and lung (left) and length of tibla (right) of the 8-week-old WT and *Agr2*/Tg mice fed a HFD for an additional 10 weeks (n=5). Representative figures were generated with data from at least three independent experiments. The data are presented as the mean ± SD values. **P < 0.01, ***P < 0.001 by Student's t test.

Supplemental Figure 3. Loss of AGR2 facilitates lipid metabolism resulting from suppression of FABP-1 in the liver. A. Volcano plots of differentially expressed genes between the livers of WT and $Agr2^{-/-}$ mice. **B.** Analysis of the acetyl-CoA level in the cytoplasm and mitochondria in HepG2 cells treated with AGR2 expression plasmids. **C.** Analysis of the ketone body content in the cytoplasm in the liver of the 8-week-old WT and *Agr2*/Tg mice fed a HFD for an additional 10 weeks (n=5). **D.** SDS–PAGE analysis of His-FABP1 fusion protein from E. coli BL21. Gel was stained with Coomassie brilliant blue R250. **E.** SDS–PAGE analysis of GST, GST-AGR2-C81A, GST-$AGR2^{WT}$, GST-$AGR2^{81\text{-}175}$ and GST-$AGR2^{101\text{-}175}$ fusion protein from E. coli BL21. Gel was stained with Coomassie brilliant blue R250. **F.** Co-immunoprecipitation

analysis of the interaction between AGR2 and ACSL5 using Huh7 cell lysates. **G.** Analysis of the acetyl-CoA level in the cytoplasm in HepG2 cells treated with AGR2 expression plasmids and siRNA targeting ACSL5. Representative figures were generated with data from at least three independent experiments. The data are presented as the mean ± SD values. *P < 0.05, **P < 0.01 by Student's t test.

Supplemental Figure 4. AGR2 influences FA absorption in the intestine. A. Flow cytometry analysis of lipid absorption in 293T cells treated with AGR2 and AGR2-C81A expression plasmids. **B.** Lipid staining in SW480 treated with siRNA targeting AGR2. **C.** Lipid staining in HT29 treated with AGR2 and AGR2-C81A expression plasmids. **D.** Lipid absorption gene expressions in the intestine of WT and *Agr2*$^{-/-}$ mice fed a NCD (n=3). **E.** Immunohistochemical staining of FABP1 and FABP2 in liver of WT and *Agr2*$^{-/-}$ mice fed a NCD. **F.** Immunohistochemical staining of FABP1 and FABP2 in liver of WT and *Agr2*/Tg mice fed a NCD. **G.** HT29 cells were treated with 10 μM CHX for 0, 2, 4, 8 and 12 h. FABP2 was detected by western blotting. Quantification was performed by normalizing proteins to β-actin. **H.** Co-immunoprecipitation analysis of the interaction between AGR2 and FABP2 using HT29 cell lysates. Representative figures were generated with data from at least three independent experiments. The data are presented as the mean ± SD values. *P < 0.05 by Student's t test.

Supplemental Figure 1

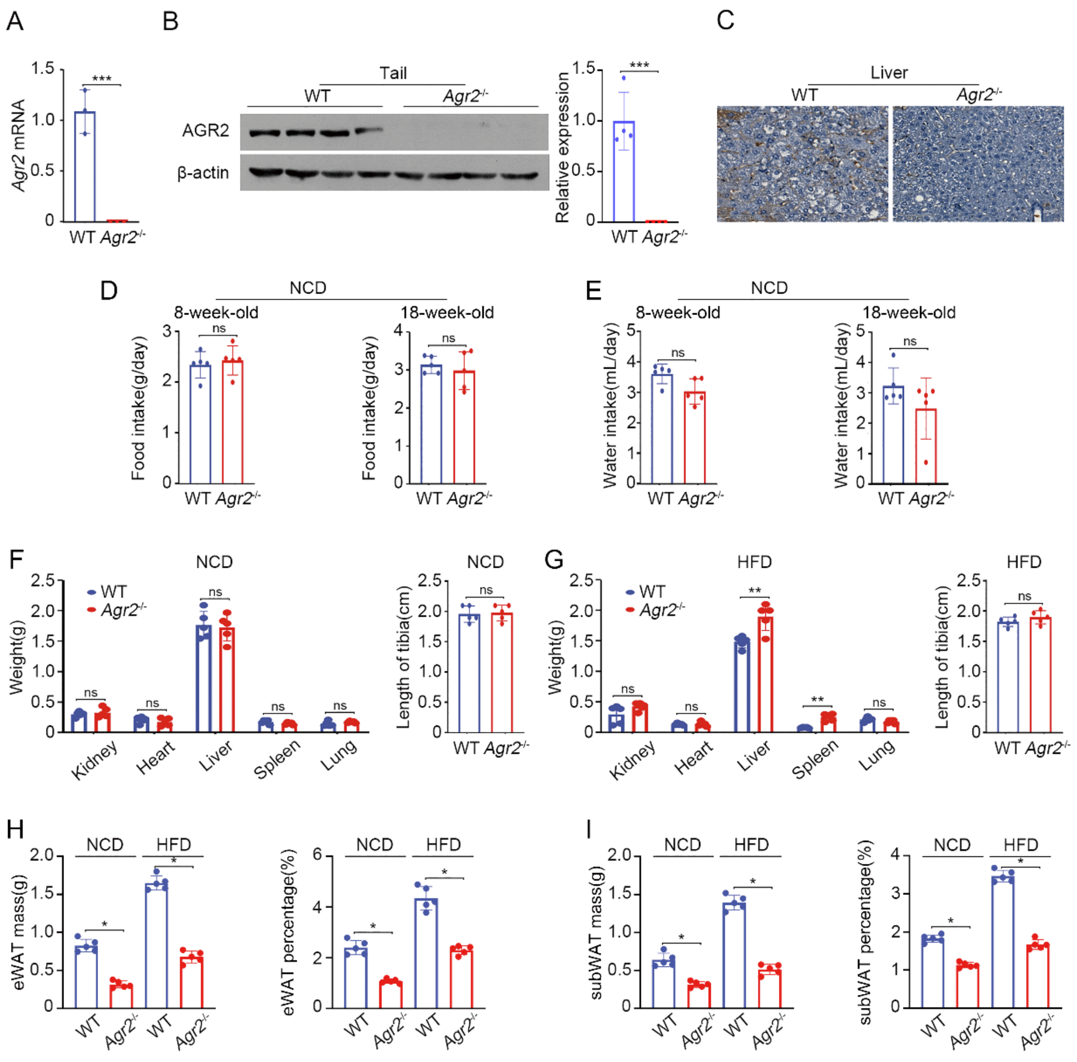

Supplemental Figure 2

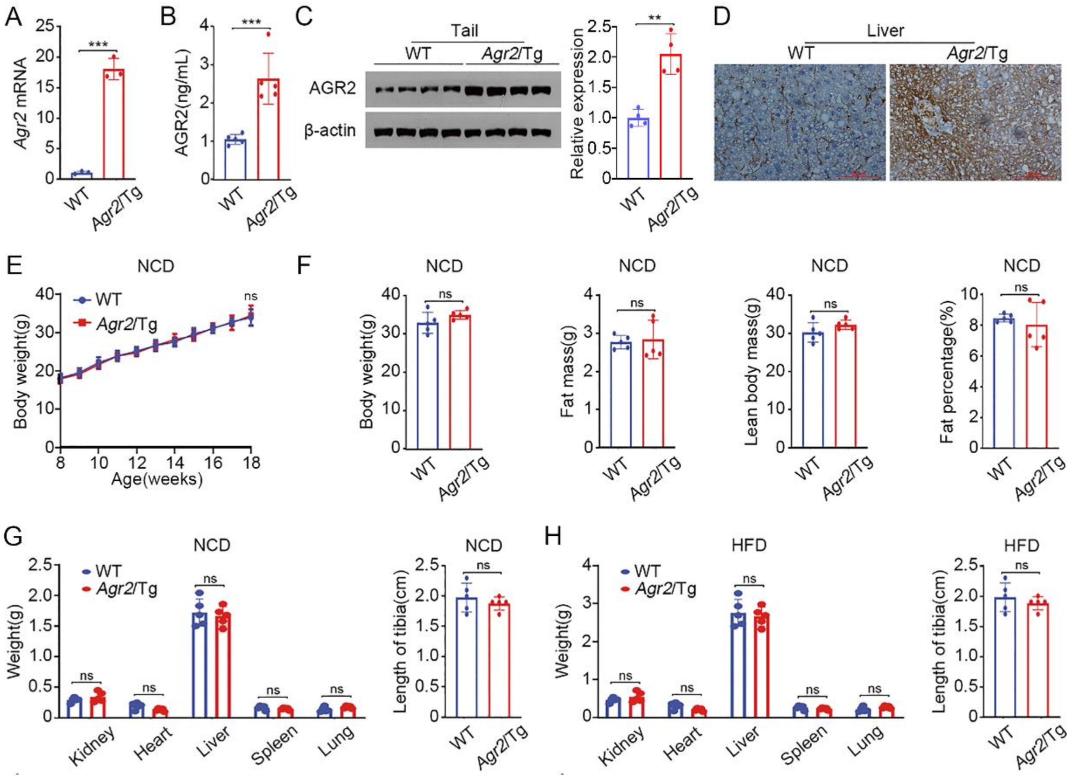

Supplemental Figure 3

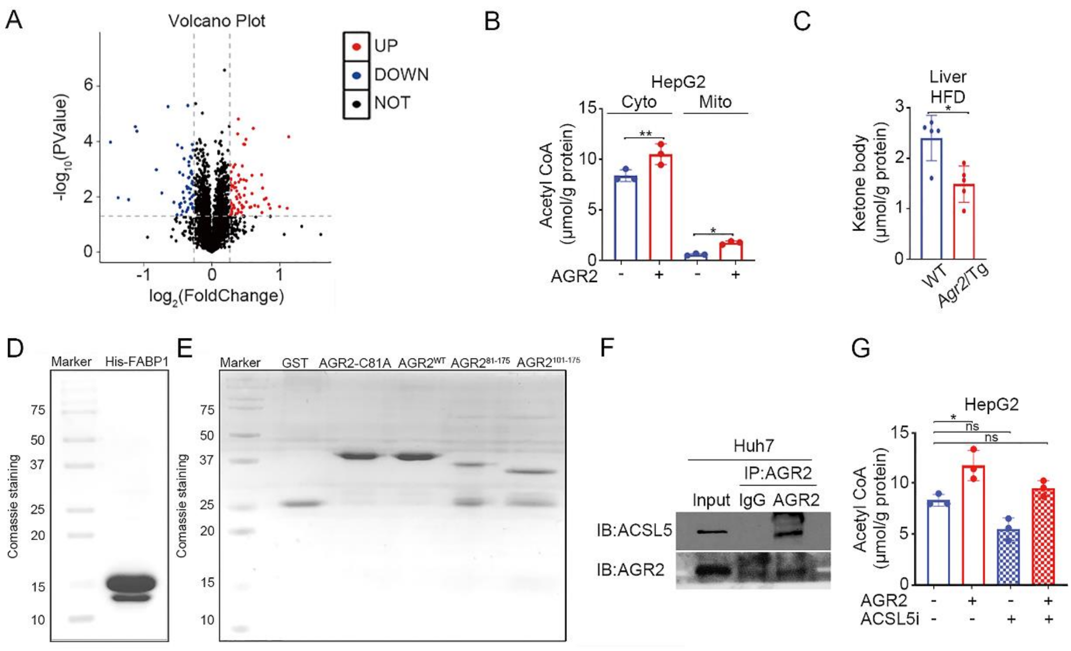

Supplemental Figure 4

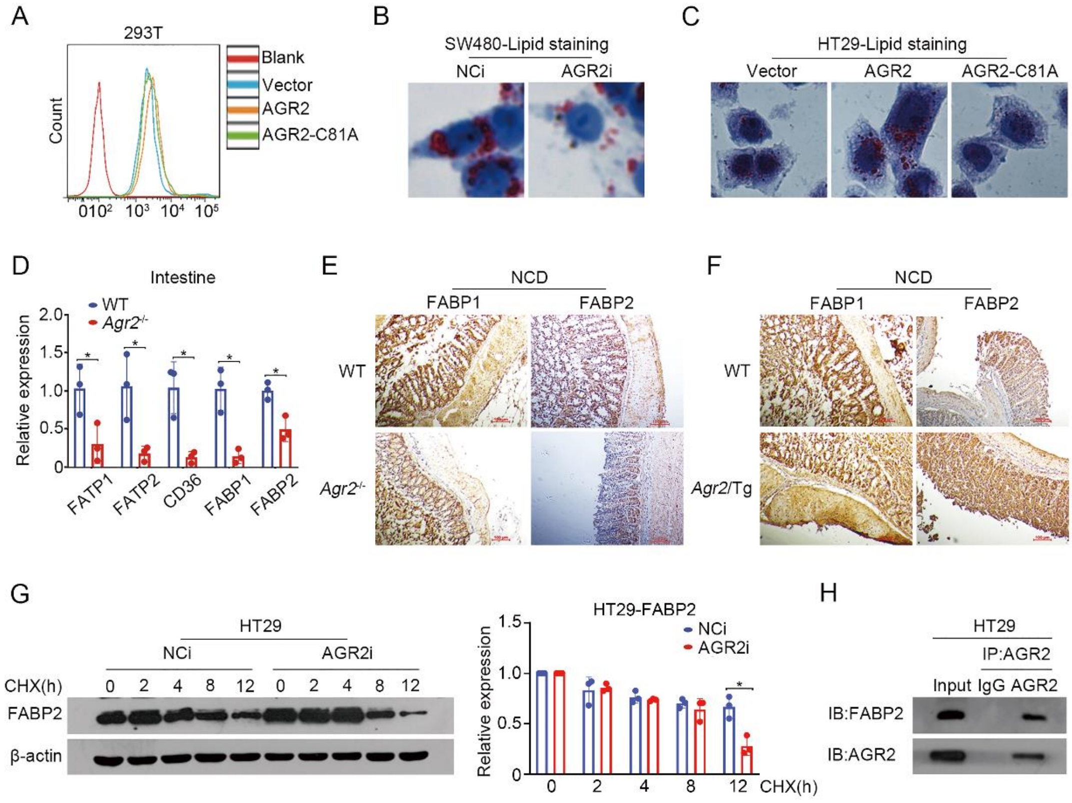

Supplement: Supplementary file 1 — Supplementary figures. [file ijbsv17p0834s1.pdf]
